# Complete Genome Sequence of *Streptococcus pneumoniae* Serotype 19A, a Blood Clinical Isolate from Northeast Mexico

**DOI:** 10.1128/genomeA.00195-16

**Published:** 2016-03-31

**Authors:** Antonio Ali Perez-Maya, Rosa Maria Hinojosa-Robles, Jose Ramon Barcenas-Walls, Armando Vignau-Cantu, Hugo A. Barrera-Saldaña, Rocio Ortiz-Lopez

**Affiliations:** a Departamento de Bioquímica y Medicina Molecular, Facultad de Medicina, Universidad Autónoma de Nuevo León, Monterrey, Nuevo León, Mexico; b Hospital Universitario Jose Eleuterio Gonzalez, Universidad Autónoma de Nuevo León, Monterrey, Nuevo León, Mexico; c Unidad de Genómica, Universidad Autónoma de Nuevo León, Centro de Investigación y Desarrollo en Ciencias de la Salud, Monterrey, Nuevo León, Mexico

## Abstract

We report here the draft genome sequence of a *Streptococcus pneumoniae* strain isolated in Monterrey, Mexico, MTY1662SN214, from a man with purpura fulminans. The strain belongs to the invasive and multidrug-resistant serogroup 19A, sequence type 320 (ST320). The draft genome sequence consists of 60 large contigs, a total of 2,069,474 bp, and has a G+C content of 39.7%.

## GENOME ANNOUNCEMENT

After the introduction of the 7-valent pneumococcal conjugate vaccine (PCV7) in the United States and other western countries, there was a significant reduction in the incidence of invasive pneumococcal disease (IPD) (1, 2). However, the number of cases of IPD, caused by *Streptococcus pneumoniae* serotypes not included in PCV7 (2, 3), increased. Particularly, the serotype 19A-sequence type 320 (ST320) has emerged worldwide as an important pathogen associated with IPD and multidrug resistance (MDR), but the causal factors remain unclear (4–6).

*S. pneumoniae* 19A ST320 has been implicated in both IPD and noninvasive pneumococcal disease (6). ST320 19A is a double-locus variant of the originally described Taiwan19F-14 (ST236) clone with high antibiotic resistance, which was isolated from a Taiwanese hospital in 1997. This strain underwent capsular modification, likely through recombination that altered the *wzy* polymerase gene and gave rise the serotype 19A-ST320 (7, 8).

*S. pneumoniae* strain MTY1662SN214 was isolated from a blood sample in 2014 at the Dr. José Eleuterio González University Hospital, Universidad Autónoma de Nuevo León (UANL), in Northeast Mexico. The patient was a 43-year-old male with purpura fulminans. The strain belonged to serotype 19A and ST320. Strain MTY1662SN214 was multidrug resistant, with resistance to penicillin, erythromycin, trimethoprim-sulfamethoxazole, tetracycline, quinolone, clindamycin, and cefotaxime (9).

The genome sequence was determined using Illumina MiSeq platform, which generated 487,316 total reads (483,492 reads after trimming) with an average length of 250 × 2 nucleotides (nt), providing about 25-fold genome coverage. *De novo* assemblies were performed by the SPAdes Genome Assembler version 3.5.0 software (10). The unclosed draft genome of MTY1662SN214 was assembled into 60 contigs (≥500 bp), with a total length of 2,069,474 bp and a G+C content of 39.7%.

In order to obtain a functional annotation of the genome, all the assembled contigs were annotated by NCBI Prokaryotic Genome Annotation Pipeline (11). The genome possesses a total of 2,221 genes, including 2,073 coding sequences (CDSs), 5 rRNAs (three copies of 5S, one copy of 16S, and one copy of 23S rRNA genes), 47 tRNAs, and 95 pseudogenes.

### Nucleotide sequence accession numbers.

This whole-genome shotgun project has been deposited at DDBJ/EMBL/GenBank under the accession no. LKAA00000000. The version described in this paper is version LKAA01000000.
